# The cost of a pediatric neurocritical care program for traumatic brain injury: a retrospective cohort study

**DOI:** 10.1186/s12913-017-2768-0

**Published:** 2018-01-12

**Authors:** Steven W. Howard, Zidong Zhang, Paula Buchanan, Stephanie L. Bernell, Christine Williams, Lindsey Pearson, Michael Huetsch, Jeff Gill, Jose A. Pineda

**Affiliations:** 1 Saint Louis University, Health Management and Policy, Salus Center 374, 3545 Lafayette Ave., St. Louis, MO 63104 USA; 2 Saint Louis University, School of Medicine, St. Louis, MO USA; 3 Saint Louis University, Center for Outcomes Research, St. Louis, MO USA; 4 Oregon State University, School of Social and Behavioral Health Sciences, Corvallis, OR USA; 5 United Healthcare, Minneapolis, MN USA; 6 St. Luke’s Health System, Kansas City, MO USA; 7 Goldfarb School of Nursing at Barnes-Jewish College, St. Louis, MO USA; 8 Division of Biostatistics, Washington University in St. Louis, School of Medicine, St. Louis, MO USA; 9 Washington University in St. Louis, School of Medicine, St. Louis, MO USA; 10 Department of Government, American University, Washington DC, USA

**Keywords:** Traumatic brain injury, Child health, Cost of care, Guidelines, Neurocritical care

## Abstract

**Background:**

Inpatient care for children with severe traumatic brain injury (sTBI) is expensive, with inpatient charges averaging over $70,000 per case (Hospital Inpatient, Children Only, National Statistics. Diagnoses– clinical classification software (CCS) principal diagnosis category 85 coma, stupor, and brain damage, and 233 intracranial injury. Diagnoses by Aggregate charges [https://hcupnet.ahrq.gov/#setup]). This ranks sTBI in the top quartile of pediatric conditions with the greatest inpatient costs (Hospital Inpatient, Children Only, National Statistics. Diagnoses– clinical classification software (CCS) principal diagnosis category 85 coma, stupor, and brain damage, and 233 intracranial injury. Diagnoses by Aggregate charges [https://hcupnet.ahrq.gov/#setup]). The Brain Trauma Foundation developed sTBI intensive care guidelines in 2003, with revisions in 2012 (Kochanek, Carney, et. al. PCCM 3**:**S1-S2, 2012). These guidelines have been widely disseminated, and are associated with improved health outcomes (Pineda, Leonard. et. al. LN 12:45-52, 2013), yet research on the cost of associated hospital care is limited. The objective of this study was to assess the costs of providing hospital care to sTBI patients through a guideline-based Pediatric Neurocritical Care Program (PNCP) implemented at St. Louis Children’s Hospital, a pediatric academic medical center in the Midwest United States.

**Methods:**

This is a retrospective cohort study. We used multi-level regression to estimate pre−/post−implementation effects of the PNCP program on inflation adjusted total cost of in-hospital sTBI care. The study population included 58 pediatric patient discharges in the pre-PNCP implementation group (July 15, 1999 - September 17, 2005), and 59 post-implementation patient discharges (September 18, 2005 - January 15, 2012).

**Results:**

Implementation of the PNCP was associated with a non-significant difference in the cost of care between the pre- and post-implementation periods (e^β^ = 1.028, *p* = 0.687).

**Conclusions:**

Implementation of the PNCP to support delivery of guideline-based care for children with sTBI did not change the total per-patient cost of in-hospital care. A key strength of this study was its use of hospital cost data rather than charges. Future research should consider the longitudinal post-hospitalization costs of this approach to sTBI care.

## Background

Severe traumatic brain injury (sTBI) is one of the most common causes of death and disability among children [1, 2]. In-hospital treatment for children with sTBI adds significant cost to the healthcare system [3], and includes intensive care in the Pediatric Intensive Care Unit (PICU) followed by medical care in the hospital ward prior to discharge from the hospital. Intensive care in the PICU focuses on reducing secondary insults that may aggravate the initial injury, including cerebral ischemia (e.g., perfusion deficits associated with dysregulated blood flow, intracranial hypertension or low blood pressure), cerebral hypoxia (e.g., diminished blood oxygen content from respiratory failure or anemia), and increased cerebral oxygen demand associated with seizures or fever [4]. Evidence-based protocols reduce the incidence of these secondary injuries and improve patient outcomes [5–9]. Although current evidence supports the adoption of Brain Trauma Foundation (BTF) guidelines, the guidelines have not been widely adopted. Providers may object for a variety of reasons including, but not limited to: difficulty in coordinating guideline implementation among subspecialties, differences between administrative and clinician cultures, physician disagreement about credibility of the evidence, and differences in physician judgment about individual patient care [10–14].

### sTBI guidelines and cost of care

Analyses of issues related to cost and BTF guidelines have generated mixed results. Qualitative work from the Commonwealth Fund suggests that the cost of providing care post-guideline implementation influences successful implementation [11]. One hospital in the Commonwealth Fund’s case study experienced a cost increase of 50% after implementing BTF guidelines [11]. The Palmer et al. study, in which the cost of providing care for thirty-seven patients treated before implementation of the BTF guidelines was compared with the cost of the 56 patients treated after implementation, came to a similar conclusion; hospital charges increased by $97,000 after guideline implementation [15]. In contrast, a recent study by Graves et al. [16] examined 235 pediatric sTBI cases across 5 regional trauma centers and found that adherence to the BTF TBI guidelines is not associated with pediatric hospital and intensive care unit (ICU) costs estimated from charges.

Notwithstanding the foundation that the aforementioned studies provide, there are important limitations. First, the focus of most of these studies is the general trauma patient population [7, 8, 10, 11, 15, 17], hence, the results may not be generalizable to childhood sTBI guideline implementation. Second, these empirical analyses use hospital charges, or cost-to-charge ratio data, as a proxy for cost of care [7, 11, 15–17]. Charges for similar services vary tremendously across hospitals. Further, hospitals negotiate discounts with insurers and work off a pre-determined fee schedule for Medicare and Medicaid patients. Therefore, using charge data as a proxy for costs can lead to erroneous analytic results [11, 18]. One of the most commonly used cost-to-charge ratios is from the HCUP dataset [16]. While useful, it oversimplifies cost of care. Each hospital in the HCUP dataset supplies a single ratio to represent the overall operations of the hospital. It is widely understood that some service lines are more profitable than others, and the cost-to-charge ratio does not permit accurate estimates of cost for individual service lines or subsets of the hospital. We sought to address this limitation in our study. In our study, St. Louis Children’s Hospital uses a modified version of activity-based costing. The cost of any specifically documented direct supplies and drugs is represented as the actual acquisition cost averaged over the fiscal reporting period. All other direct costs are allocated based on time-studies.

This study aims to examine the actual cost of providing inpatient care to children with sTBI before and after implementation of a Pediatric Neurocritical Care Program (PNCP) that supported care based on the BTF guidelines. The study’s objective was driven by the Institute for Healthcare Improvement’s Triple Aim – to improve health outcomes and enhance the patient care experience while controlling costs [19, 20]. The setting is St. Louis Children’s Hospital (Missouri, USA), a regional university-affiliated hospital with 280 beds and a Level 1 trauma center. The PNCP at St. Louis Children’s Hospital is imbedded in the Pediatric Intensive Care Unit and supports multidisciplinary care provided by specialists with board certification in pediatric critical care medicine, neurosurgery, neurology, neuroradiology and general surgery. The program’s medical director and a full time clinical nurse coordinator facilitate multidisciplinary implementation of care pathways by facilitating coordinated decision making between services, supporting time-sensitive and goal-directed interventions. Research procedures were approved and exempted from informed consent by the Washington University Institutional Review Board.

An earlier study by Pineda et al. reported decreased mortality and increased probability of favorable disposition at hospital discharge at St. Louis Children’s Hospital (SLCH) after implementing an approach to care based on the BTF guidelines [21]. This approach was associated with increased use of medical therapies (i.e., earlier and more intense monitoring and treatment of increased intracranial pressure) included in the BTF guidelines. Details of the PNCP pathway of care for sTBI are detailed elsewhere [21]. Based on work by March [11], and Palmer et al. [15], we hypothesized that supporting implementation of the BTF guidelines for pediatric sTBI care would be associated with higher costs of hospital care.

## Methods

In this retrospective cohort study, we screened all patients admitted to the PICU at SLCH with sTBI, defined by a Glasgow Coma Scale (GCS) score of 8 or less after resuscitation in the Emergency Department [16, 22]. Due to significant differences in pathophysiology and mortality, patients with GCS score of 3, bilateral fixed and dilated pupils on admission to the Emergency Department, cardiac arrest prior to admission to the pediatric intensive care unit (PICU), abusive head trauma, or gunshot wounds to the head were excluded from the analysis [23, 24]. As the main purpose of the study was to evaluate the impact of the PNCP approach to sTBI care on hospital cost, patients with early death (<48 h) were excluded from the study [16, 21]. After exclusions, we analyzed the cost data for 124 patients admitted to the SLCH PICU between July 15, 1999 and January 15, 2012. Of the 124 patients, SLCH discharged 64 patients before September 17, 2005 and these subjects were included in the pre-PNCP implementation group. SLCH discharged another 60 patients on or after September 17, 2005 and they were included in the post-implementation group.

Medical chart and demographic data were merged with the hospital’s cost of care data for the same patient hospitalizations. Inpatient costs were inflation-adjusted to 2012 dollars using the Medical Commodity Consumer Price Index (CPI) and Medical Services CPI [25]. Upon examination of the data, we excluded a total of 7 subjects from the analysis due to either missing data (1 pre, 0 post), total costs more than 3 standard deviations from the mean (0 pre, 1 post (whose total cost exceeded10 standard deviations above the mean)) [26–29], or who died within the first 48 h after admission (5 pre, 0 post). The final analytic sample contained 117 pediatric patients (58 pre and 59-post PNCP implementation).

A multilevel regression model adjusted for the effects of patients’ demographic and clinical characteristics on total cost, and random effects were incorporated to account for unobserved changes over time (covariates were nested within groupings of years: 1999–2002, 2003–2005, 2006–2008, and 2009–2012, with a similar number of subjects in each). To meet the assumption of normality, we log-transformed the total cost outcome variable [30–32]. Demographic and clinical factors included patient sex, race, age at admission (years), mechanism of injury, lengths of stay (LOS) in days in the pediatric intensive care unit (PICU) and the hospital’s general ward (floor LOS). We represented the severity of TBI and risk of death for each patient with Glasgow Coma Score (GCS) and Pediatric Risk of Mortality (PRISM III) scores, respectively [5, 33]. The GCS scale ranges from 3 to 15, and a score of 8 or less after resuscitation in the Emergency Department represents sTBI [5]. The PRISM III score is measured in the first 12 h after admission to the PICU and reliably predicts risk of mortality in critically ill children, with higher scores representing higher risk of mortality [33]. We merged clinical data with cost data from the hospital’s finance office. To assure patient and facility confidentiality (as approved by the IRB), we converted the cost per patient from actual dollars to the “percent of pre-PNCP patient mean total cost”. For example, a patient whose total cost was exactly equal to the mean of pre-PNCP patients’ costs is coded as 100.0 [25]. A cost code of 105 signifies that a patient had total costs 5% higher than pre-PNCP average costs. We also analyzed subcategories of cost relevant to TBI care in the PICU for which financial data was available.

Descriptive analysis was performed for the pre-PNCP and post-PNCP groups separately, and statistics are presented in Table 1. Bivariate analysis included chi-squared tests for dichotomous variables, and for continuous variables, we used the Wilcoxon test for medians, and *t*-test for means. As in prior cost studies, we used multi-level linear regression to model the effect of the PNCP program’s implementation on the total cost outcome variable (Table 2) and address the random effect of yearly variation [34]. The analysis of service type use and cost is presented in Table 3. SAS v.9.4 was used for all analyses, with significance level set at 0.05.

**Table 1:** Descriptive statistics by PNCP implementation status (*N* = 117)

Variable	Before PNCP implementation (*n* = 58) N (%) or mean +/− SD	After PNCP implementation (*n* = 59) N (%) or mean +/− SD)	*p*-value
Sex (male)	32 (55%)	33 (56%)	0.934
Race (non-white)	14 (24%)	17 (29%)	0.567
Injury mechanism (motor vehicle)	36 (62.1%)	41 (69.5%)	0.397
Age at admission (years)			
Mean (SD)	11.0 (4.7)	11.9 (4.5)	0.271
Median (IQR)	12.1 (8.4–14.6)	12.4 (8.7–16.2)	0.250
--- 0 to 5	9 (15.5%)	8 (13.6%)	0.601
--- 6 to 11	19 (32.8%)	19 (32.2%)	
--- 12 to 15	20 (34.5%)	16 (27.1%)	
--- 16 to 18	10 (17.2%)	16 (27.1%)	
Glasgow Coma Score (GCS)^a^			
Mean (SD)	5.1 (1.8)	4.5 (1.6)	0.041
Median (IQR)	5.0 (3.0–7.0)	4.0 (3.0–6.0)	0.047
GCS 3–5 N (%)	23 (39.7%)	33 (55.9%)	0.078
GCS >5 N (%)	35 (60.3%)	26 (44.1%)	
Pediatric Risk of Mortality Score (PRISM III)			
Mean (SD)	5.4 (4.3)	5.9 (5.8)	0.557
Median (IQR)	4.5 (2.0–9.0)	5.0 (2.0–8.0)	0.928
1st Quartile N (%)	6 (10.3%)	9 (15.3%)	0.877
2nd Quartile N (%)	23 (39.7%)	16 (27.1%)	
3rd Quartile N (%)	14 (24.1%)	22 (37.3%)	
4th Quartile N (%)	15 (25.9%)	12 (20.3%)	
PICU length of stay (days)			
Mean (SD)	12.0 (7.9)	14.7 (9.0)	0.090
Median (IQR)	12.0 (6.0–15.0)	14.0 (7.0–22.0)	0.097
1st Quartile N (%)	10 (17.2%)	8 (13.6%)	0.144
2nd Quartile N (%)	18 (31.0%)	15 (25.4%)	
3rd Quartile N (%)	19 (32.8%)	16 (27.1%)	
4th Quartile N (%)	11 (19.0%)	20 (33.9%)	
General/Floor length of stay (days)			
Mean (SD)	33.7 (33.4)	30.3 (28.7)	0.549
Median (IQR)	26.0 (7.0–42.0)	22.0 (8.0–38.0)	0.664
1st Quartile N (%)	14 (24.1%)	11 (18.6%)	0.700
2nd Quartile N (%)	11 (19.0%)	19 (32.2%)	
3rd Quartile N (%)	16 (27.6%)	15 (25.4%)	
4th Quartile N (%)	17 (29.3%)	14 (23.7%)	
Overall hospital length of stay (days)			
Mean (SD)	45.8 (38.3)	45.0 (34.1)	0.908
Median (IQR)	34.0 (17.0–56.0)	37.0 (17.0–58.0)	0.946
Total inpatient cost percentage^b^	100 (78.0)	107 (72.4)	0.607
Mean (SD)	100.0 (78.0)	107.2 (72.4)	0.607
Median (IQR)	67.6 (46.9–119.6)	88.7 (57.5–132.2)	0.377

^a^GCS score < 8 is considered sTBI. GCS scores in the study population ranged between 3 and 8

^b^Percentage is based on mean total cost pre-PNCP

PNCP - Pediatric Neurocritical Care Program

PICU – Pediatric Intensive Care Unit

**Table 2:** Total cost regression model^a^

Variable	e(β)	*p*-value
Post-PNCP Implementation	0.933 (0.722, 1.205)	0.594
Sex (male)	0.992 (0.903, 1.090)	0.869
Race (non-white)	1.007 (0.904, 1.121)	0.902
Age at admission (years)		
Age 0–5 years	(reference)	
Age 6–11 years	1.061 (0.920, 1.224)	0.416
Age 12–15 years	1.062 (0.921, 1.223)	0.407
Age 16–18 years	1.104 (0.949, 1.284)	0.199
Injury mechanism (non-motor vehicle)	0.963 (0.872, 1.063)	0.452
Glasgow Coma Score (GCS > 5)^a^	0.936 (0.853, 1.028)	0.165
Pediatric Risk of Mortality Score (PRISM III)		
2nd Quartile	1.030 (0.891, 1.190)	0.691
3rd Quartile	1.016 (0.879, 1.173)	0.832
4th Quartile	1.026 (0.878, 1.200)	0.746
PICU LOS		
1st Quartile (vs. cost for 2nd quartile LOS)	0.543 (0.471, 0.627)	<0.001
3rd Quartile (vs. cost for 2nd quartile LOS)	1.413 (1.251, 1.596)	<0.001
4th Quartile (vs. cost for 2nd quartile LOS)	2.091 (1.827, 2.392)	<0.001
General/Floor LOS		
1st Quartile N (vs. cost for 2nd quartile LOS)	0.714 (0.622, 0.820)	<0.001
3rd Quartile N (vs. cost for 2nd quartile LOS)	1.311 (1.157, 1.486)	<0.001
4th Quartile N (vs. cost for 2nd quartile LOS)	2.143 (1.872, 2.454)	<0.001

^a^estimate for GCS is for score of 6–8 category vs. reference group of 3–5

*PNCP* Pediatric Neurocritical Care Program

*GCS* Glasgow Coma Score

*PRISM* Pediatric Risk of Mortality Score

*PICU* Pediatric Intensive Care Unit

*LOS* Length of Stay

**Table 3:** Descriptive statistics of services provided before and after PNCP implementation status (*N* = 117)

	Utilization per Patient			% Change in Cost per Item		% Change in Total Cost per Patient	
Service Type	Pre-PNCC Mean/SD (N out of 58)	Post-PNCC Mean/SD (N out of 59)	*p*-value	% change from Pre-PNCC	*p*-value	% change from Pre-PNCC	*p*-value
Admission CT^‡^ scans per patient	0.95/0.22 (55)	0.97/0.32 (55)	0.727	−41%	0.001*	−40%	0.001*
Subsequent CT scans per patient	2.62/2.43 (51)	2.24/1.76 (48)	0.331	−36%	0.001*	−46%	0.018*
End Tidal CO_2_ (days monitored)	7.98/6.65 (52)	11.37/7.82 (57)	0.013*	−6%	0.001*	+47.8%	0.005*
ICP Monitoring (days monitored)	2.59/3.02 (35)	4.44/5.49 (42)	0.026*	−8%	0.002*	+57.4%	0.059
External Ventricular Drains (EVD) (ave.# procedures per patient)	0.21/0.41 (12)	0.05/0.22 (3)	0.013*	(not available)		(not available)	
Hyperosmolar Therapy: 3% Hypertonic Saline or Mannitol (doses per patient)	4.83/5.70 (42)	8.46/8.26 (52)	0.007*	+22%	0.027*	+77.8%	0.056
Pentobarbital (doses per patient)	0.43/0.80 (17)	1.66/3.24 (19)	0.006*	+2325%	0.001*	+13,638%	0.005*
Epinephrine (infusions per patient)	0.09/0.28 (5)	0.10/0.36 (5)	0.796	+30%	0.430	+155.9%	0.291
Norepinephrine (infusions per patient)	(no utilization)	0.53/1.44 (9)	n/a	(no pre-PNCC utilization)	n/a	(no pre-PNCC utilization)	n/a
Dopamine (infusions per patient)	1.48/3.09 (19)	1.63/2.89 (23)	0.795	−14%	0.567	+18.8%	0.810
Fentanyl (doses per patient)	8.17/6.94 (56)	8.86/5.31 (59)	0.546	+129%	0.001*	+361.7%	<0.001*
Midazolam (doses per patient)	7.64/5.85 (57)	9.95/6.07 (56)	0.038*	−73%	0.001*	−34.8%	0.262
Morphine (doses per patient)	2.55/2.99 (34)	12.24/12.62 (49)	0.001*	−28%	0.129	+363.5%	0.020*
Dexmedetomidine (infusions per patient)	(no utilization)	2.14/3.46 (29)	n/a	(no pre-PNCC utilization)	n/a	(no pre-PNCC utilization)	n/a
sTBI-care labs (lab tests per patient)^†^	56.83/39.28 (58)	69.88/42.37 (59)	0.087	−6%	0.023*	+16.0%	0.272
Sodium monitoring (ave.# tests per patient)	0.45/0.50 (26)	0.54/0.50 (32)	N/S	(not available)		(not available)	
Osmolality monitoring (ave.# tests per patient)	0.72/0.45 (42)	0.78/0.42 (46)	N/S	(not available)		(not available)	
EEG monitoring (ave.# tests per patient)	0.22/0.42 (13)	0.31/0.46 (18)	N/S	(not available)		(not available)	

*p-values below 0.05 indicate service type encountering statistical significance

†See Table 4 for details about sTBI-care labs

‡Admission CT per patient mean is <1.0 because some patients receive initial CT from referring hospitals

*PNCP* Pediatric Neurocritical Care Program; *CT* computerized tomography; *ICP* intracranial pressure; *sTBI* severe traumatic brain injury

## Results

### Descriptive statistics (table 1)

The differences in the quartile distributions of PICU LOS pre- and post-implementation, though not statistically significant (*p* = 0.144), appear to show more patients with higher LOS after PNCP implementation. Lengths of stay in the general ward, or hospital floor, and overall hospital LOS were slightly shorter post-implementation, but again, the differences were not significant (*p* = 0.549 and 0.908, respectively). We observed slightly lower GCS scores post-PNCP implementation (4.5, vs. 5.1 pre-implementation) suggesting more severe TBI in the post-PNCP group. While small and not clinically meaningful, this change was statistically significant (*p* = 0.041). There was no statistically significant difference in the mean total cost of care, from pre- to post-implementation of the PNCP program (*p* = 0.607). Furthermore, there was no significant difference in the distribution of total cost of care (*p*-value of difference in medians = 0.377).

### Regression model

Table 2 presents the results of the Total Cost regression model. After adjusting for the covariates presented in Table 1 (using categorized PICU and floor LOS), PNCP implementation was not significantly associated with total cost of care (e^B^ = 0.933, *p* = 0.594). Both PICU LOS and hospital floor LOS were significant, with predictably higher costs associated with longer lengths of stay (as measured by quartiles of LOS). Patients with PICU or floor LOS in the 4th quartile were more than twice as costly as those in the 2nd quartile reference group. To evaluate whether the observation excluded on the basis of high cost could be an influential outlier, we ran the model again with it included. There was no change in magnitude, direction, or significance of the model estimates.

### Subcategory analysis

Hospital cost and utilization data were available for 14 products and services related to sTBI care. Utilization of products and services used in treating sTBI either did not change significantly or increased in the post-PNCP period (Table 3). There was a significant reduction in cost per item for six of the fourteen services. The unit costs of three services significantly increased and three remained statistically unchanged. For two services, pre-PNCP utilization and cost data were not available.

## Discussion

Although longer LOS in the PICU and on the floor are associated with increased costs, holding all else constant, the PNCP program did not result in increased costs. Previous findings attributed this increase in LOS to increased survival rates - lives previously lost to sTBI were more likely to be saved, but to require more time in the PICU [21]. We surmise that the magnitude of the post-implementation decrease in LOS on the floor was enough to counter the post-implementation increased LOS in the PICU (though neither had statistically significant effects).

As expected, supporting care based on the BTF guidelines increased utilization of products and services related to TBI care. As noted in Table 3, there was an increase in intracranial pressure (ICP) monitoring post-PNCP. We acknowledge that length of ICP monitoring is influenced by increased survival, but importantly, it still represents increased cost to the healthcare system. There was also increased utilization of medications used to treat intracranial hypertension (hyperosmolar therapies, pentobarbital, midazolam and morphine). Fentanyl is also used to treat intracranial hypertension, but utilization did not change. Dexmedetomidine is a sedative used to reduce agitation that may lead to intracranial hypertension, but was not commercially available pre-PNCP. The guidelines also recommend optimizing blood pressure to maintain adequate cerebral perfusion pressure, but do not specify the type of medication used to achieve this goal. While Norepinephrine was introduced post-PNCP, likely reflecting practice preferences, overall there was no change in utilization of medications used to maintain adequate blood pressure (Epinephrine and Dopamine).

The guidelines also recommend avoiding hyperventilation, which requires continuous monitoring of exhaled CO_2_ (End Tidal CO_2_ monitoring). We found that End Tidal CO_2_ monitoring utilization increased post-PNCP, suggesting increased compliance with the guidelines. However, retrospectively, we cannot rule out that this increase was simply a consequence of increased length of mechanical ventilation (data on cost of mechanical ventilation was unavailable for too many pre-PNCP patients, precluding inclusion in our analysis). As is the case of length of ICP monitoring, length of End Tidal CO_2_ monitoring in the ICU may also be influenced by survival.

Finally, there was an increase in the utilization of laboratory studies related to sTBI care (for a complete list, see online supplemental Table 4). This finding, however, was not statistically significant. Table 3 also includes data on computed tomography (CT) imaging. While there was no change in utilization of CT scans, reductions in cost per scan resulted in overall lower costs of CT scans per patient.

**Table 4:** Descriptions of 21 sTBI-care Labs

Charge Description	ᅟ
Basic Metabolic Panel	
CBC Auto with Auto Diff	
CBC Auto without Auto Diff	
Gases Blood Any Combination	
Osmolality	
Gases Blood with O_2_ Sat	
Thromboplastin Time Portal	
Prothrombin Time	
Sodium Body Fluid	
Sodium	
Comp Metabolic Panel	
Blood Count Hemoglobin	
Sodium Urine	
Osmolality Urine	
Electrolyte Panel	
Glucose	
CBC with Manual Diff	
Blood Gas	
Cortisol Total	
Renal Function Panel	
Hematocrit	

Our results are encouraging for provider organizations that seek to improve outcomes in the wake of economic constraints. Together with the findings of improved patient outcomes by Pineda et al. and others [7, 11, 21], this analysis supports implementation of the BTF guidelines in clinical practice. Importantly, the PNCP supported this approach to care without requiring creation of a separate service or intensive care unit, making such approach to care easier to reproduce [35]. As the global impact of neurological diagnoses in critically ill children and the approaches to pediatric neurocritical care continue to be better defined [36–39] cost of implementation will become easier to assess and compare.

A central strength of our study was its pre/post design and use of inflation-adjusted hospital cost data rather than hospital charges. This addressed a key limitation of previous studies that used only hospital charges to approximate the economic effects of BTF guideline implementation [7, 11, 15–17]. In addition, this is the first research into the utilization changes for specific products and services related to sTBI care after guideline implementation.

This research has limitations. The data were restricted to only the 117 sTBI patients treated at one major academic medical center in the Midwest over 12 years. Actual costs of care also vary greatly between and within different regions of the country, and globally.

To provide some insights into the nature of the cost variations, Table 3 presents the changes in costs per item within the institution over time. It is reasonable to accept these variations in unit cost as reflecting common hospital accounting practice, introduction of new drugs, established drugs coming off-patent, and periodic changes in vendor pricing. However, in combination with utilization changes, unit cost changes can amplify or diminish reductions in total cost per patient or total cost increases (such as increase in both utilization and unit cost for pentobarbital, a medication commonly used to treat patients with sTBI).

Multilevel regression with random effects modeling was adopted to address these variations based on the assumption that the effect of periodic variation is random. Variations in cost are determined by multiple factors including accounting practices for capital equipment and contracts with vendors. Additionally, we assumed that if a service is performed, the hospital always accounts for the cost of that item. However, in our retrospective analysis, it is not possible to identify cases in which zero cost represents lack of utilization versus omission of cost data entry.

Retrospective cohort studies are also limited by changes in clinical practice over time, and it is not possible to control for all factors that may affect both clinical practice and cost. We attempted to mitigate this limitation by incorporating random effects (based on year) into our statistical model, and exploring within-center practice variability and secular trends in this cohort of patients [21]. It is also important to highlight the large amount of between-center variability in severe TBI care –multicenter studies will be needed to broadly address the economic impact of clinical practice [40–42].

Our analysis excluded patients with early deaths and a patient who was an outlier (cost over 10 standard deviations from the mean). While this approach facilitates comparison with previous studies and may more accurately reflect adherence to the BTF guidelines [17], it could also introduce selection bias. Some of these limitations may be addressed in larger, prospective studies; although, even with a larger sample, cautious interpretation is advised.

Finally, the PNCP operating costs (i.e., physician, clinical nurse coordinator, data management and overhead or fixed cost allocations) were not included in this analysis; PNCP resources are not exclusively dedicated to TBI patients, making this cost assignment difficult to quantify or reasonably allocate.

There are a number of opportunities for future research in this area. The apparent trade-off between increases in costly PICU days and reductions in less costly general inpatient floor days is ripe for future research. In addition, it would behoove researchers to explore in more detail the directions and magnitudes of cost fluctuations by category of sTBI product and service. Further, a longitudinal study of the cost of care for sTBI patients across the care continuum is needed. Such a study would be able to consider the costs to survivors, families, and society of TBI rehabilitation and disability services. Although longitudinal costs across multiple care providers were beyond the scope of the current study, it is reassuring to see that care based on recommendations from the BTF did not increase the cost of the acute initial hospitalization for these patients. To better understand the extent to which the investment in PNCP care yields cost savings to society, future work is planned to analyze total patient costs over a longer time span, including care after hospital discharge. Incorporating all trauma centers in the state will also enable the comparison of BTF guideline adopters vs. non-adopters.

## Conclusions

In a pediatric neurocritical care program (PNCP), the primary drivers of cost are lengths of stay in the PICU and on the hospital floor. This study found that implementation of the PNCP did not have a statistically significant impact on average length of stay, and did not change the total perpatient cost of in-hospital care. Taken together with the results of previous sTBI outcomes research studies, our findings support the position that healthcare managers and clinicians continue to support delivery of guideline-based care for children with sTBI. Significant improvements in patient outcomes can be achieved for little or no increase in the overall cost of inpatient care.

## Abbreviations

BTF: Brain Trauma Foundation
CO_2_: Carbon dioxide
CPI: Consumer Price Index
CT: Computed tomography
GCS: Glasgow Coma Scale/Score
ICP: Intracranial pressure
ICU: Intensive care unit
LOS: Length of stay
PICU: Pediatric intensive care unit
PNCP: Pediatric Neurocritical Care Program
PRISM: Pediatric Risk of Mortality
SLCH: St. Louis Children’s Hospital
sTBI: Severe traumatic brain injury

## References

[1] TBI: Get the Facts [http://www.cdc.gov/traumaticbraininjury/get_the_facts.html].

[2] Shi J, Xiang H, Wheeler K, Smith GA, Stallones L, Groner J, Wang Z (2009). Costs, mortality likelihood and outcomes of hospitalized US children with traumatic brain injuries. Brain Inj.

[3] Hospital Inpatient, Children Only, National Statistics. Diagnoses– clinical classification software (CCS) principal diagnosis category 85 coma, stupor, and brain damage, and 233 intracranial injury. Diagnoses by Aggregate charges. [https://hcupnet.ahrq.gov/#setup].

[4] Scaife ER, Statler KD (2010). Traumatic brain injury: preferred methods and targets for resuscitation. Curr Opin Pediatr.

[5] Kochanek PM, Carney N, Adelson PD (2012). Guidelines for the acute medical Management of Severe Traumatic Brain Injury in infants, children, and adolescents-second edition. Pediatr Crit Care Med.

[6] Dean NP, Boslaugh S, Adelson PD, Pineda JA, Leonard JR (2007). Physician agreement with evidence-based recommendations for the treatment of severe traumatic brain injury in children. J Neurosurg.

[7] Fakhry SM, Trask AL, Waller MA, Watts DD (2004). Management of brain-injured patients by an evidence-based medicine protocol improves outcomes and decreases hospital charges. J Trauma Acute Care Surg.

[8] Gerber LM, Chiu Y-L, Carney N, Härtl R, Ghajar J (2013). Marked reduction in mortality in patients with severe traumatic brain injury: clinical article. J Neurosurg.

[9] Tilford JM, Aitken ME, Anand KJ, Green JW, Goodman AC, Parker JG, Killingsworth JB, Fiser DH, Adelson PD (2005). Hospitalizations for critically ill children with traumatic brain injuries: a longitudinal analysis. Crit Care Med.

[10] Eddy DM (2005). Evidence-based medicine: a unified approach. Health Aff.

[11] March A. Commonwealth Fund: Facilitating implementation of evidence-based guidelines in hospital settings: learning from trauma centers. New York City: Citeseer; 2006. http://citeseerx.ist.psu.edu/viewdoc/download?doi=10.1.1.548.6815&rep=rep1&type=pdf.

[12] Timbie JW, Fox DS, Van Busum K, Schneider EC (2012). Five reasons that many comparative effectiveness studies fail to change patient care and clinical practice. Health Aff.

[13] Brolliar SMMM, Thompson HJ, Whiteside LK, Mink RB (2016). A qualitative study exploring factors associated with provider adherence to severe pediatric traumatic brain injury guidelines. J Neurotrauma.

[14] Roumeliotis N, Pettersen G, Crevier L, Emeriaud G (2015). ICP monitoring in children: why are we not adhering to guidelines. Childs Nerv Syst.

[15] Palmer S, Bader MK, Qureshi A, Palmer J, Shaver T, Borzatta M, Stalcup C (2001). The impact on outcomes in a community hospital setting of using the AANS traumatic brain injury guidelines. J Trauma Acute Care Surg.

[16] Graves JM, Kannan N, Mink RB, Wainwright MS, Groner JI, Bell MJ, Giza CC, Zatzick DF, Ellenbogen RG, Boyle LN (2016). Guideline adherence and hospital costs in pediatric severe traumatic brain injury. Pediatr Crit Care Med.

[17] Vitaz T, Mcilvoy L, Raque G, Spain D, Shields C (2001). Development and implementation of a clinical pathway for severe traumatic brain injury. J Trauma.

[18] Finkler SA (1982). The distinction between cost and charges. Ann Intern Med.

[19] IHI Europe [http://www.ihi.org/regions/Europe/Pages/default.aspx].

[20] Berwick DM, Nolan TW, Whittington J (2008). The triple aim: care, health, and cost. Health Aff.

[21] Pineda JA, Leonard JR, Mazotas IG, Noetzel M, Limbrick DD, Keller MS, Gill J, Doctor A (2013). Effect of implementation of a paediatric neurocritical care programme on outcomes after severe traumatic brain injury: a retrospective cohort study. Lancet Neurol.

[22] Maas AI, Harrison-Felix CL, Menon D, Adelson PD, Balkin T, Bullock R, Engel DC, Gordon W, Langlois-Orman J, Lew HL (2011). Standardizing data collection in traumatic brain injury. J Neurotrauma.

[23] Ducrocq SC, Meyer PG, Orliaguet GA, Blanot S, Laurent-Vannier A, Renier D, Carli PA (2006). Epidemiology and early predictive factors of mortality and outcome in children with traumatic severe brain injury: experience of a French pediatric trauma center. Pediatr Crit Care Med.

[24] Figaji AA, Zwane E, Thompson C, Fieggen AG, Argent AC, Le Roux PD, Peter JC (2009). Brain tissue oxygen tension monitoring in pediatric severe traumatic brain injury. Childs Nerv Syst.

[25] Consumer Price Index [http://www.bls.gov/cpi/cpifact8.htm].

[26] Frees EW (1996). Data analysis using regression models: the business perspective.

[27] Ryser DK, Egger MJ, Horn SD, Handrahan D, Gandhi P, Bigler ED (2005). Measuring medical complexity during inpatient rehabilitation after traumatic brain injury. Arch Phys Med Rehabil.

[28] Chan E, Zhan C, Homer CJ (2002). Health care use and costs for children with attention-deficit/hyperactivity disorder: national estimates from the medical expenditure panel survey. Arch Pediatr Adolesc Med.

[29] Kappelman MD, Rifas–Shiman SL, Porter CQ, Ollendorf DA, Sandler RS, Galanko JA, Finkelstein JA (2008). Direct health care costs of Crohn's disease and ulcerative colitis in US children and adults. Gastroenterology.

[30] Griswold M, Parmigiani G, Potosky A, Lipscomb J (2004). Analyzing health care costs: a comparison of statistical methods motivated by Medicare colorectal cancer charges. Biostatistics.

[31] Manning WG, Mullahy J (2001). Estimating log models: to transform or not to transform?. J Health Econ.

[32] Pagano E, Petrelli A, Picariello R, Merletti F, Gnavi R, Bruno G (2015). Is the choice of the statistical model relevant in the cost estimation of patients with chronic diseases? An empirical approach by the piedmont diabetes registry. BMC Health Serv Res.

[33] Pollack MM, Patel KM, Ruttimann UE, III PRISM (1996). An updated pediatric risk of mortality score. Crit Care Med.

[34] Carey K (2002). Hospital length of stay and cost: a multilevel modeling analysis. Health Services and Outcomes Res Methodology.

[35] O’Lynnger TM, Shannon CN, Le TM, Greeno A, Chung D, Lamb FS, JCW III (2016). Standardizing ICU management of pediatric traumatic brain injury is associated with improved outcomes at discharge. J Neurosurg Pediatr.

[36] Fink EL, Kochanek PM, Tasker RC, Beca J, Bell MJ, Clark RSB, Hutchison J, Vavilala MS, Fabio A, Angus DC, Watson RS (2017). International survey of critically ill children with acute neurologic insults: the prevalence of acute critical neurological disease in children: a global epidemiological assessment study. Pediatr Crit Care Med.

[37] Markham C, Proctor EK, Pineda JA (2017). Implementation strategies in pediatric neurocritical care. Curr Opin Pediatr.

[38] Murphy S (2012). Pediatric Neurocritical Care. Neurotherapeutics.

[39] Tasker RC (2009). Pediatric neurocritical care: is it time to come of age?. Curr Opin Pediatr.

[40] Bennett TD, Riva-Cambrin J, Keenan HT, Korgenski E, Bratton SL (2012). Variation in intracranial pressure monitoring and outcomes in pediatric traumatic brain injury. Arch Pediatr Adolesc Med.

[41] Kurz JE, Poloyac SM, Abend NS, Fabio A, Bell MJ, Wainwright MS (2016). Variation in anticonvulsant selection and electroencephalographic monitoring following severe traumatic brain injury in children—understanding resource availability in sites participating in a comparative effectiveness study. Pediatr Crit Care Med.

[42] Bennett TD, Statler KD, Korgenski EK, Bratton SL (2012). Osmolar therapy in pediatric traumatic brain injury. Crit Care Med.

